# Toward a multi-level strategy to reduce stigma in global mental health: overview protocol of the Indigo Partnership to develop and test interventions in low- and middle-income countries

**DOI:** 10.1186/s13033-022-00564-5

**Published:** 2023-02-03

**Authors:** Petra C. Gronholm, Ioannis Bakolis, Anish V. Cherian, Kelly Davies, Sara Evans-Lacko, Eshetu Girma, Dristy Gurung, Charlotte Hanlon, Fahmy Hanna, Claire Henderson, Brandon A. Kohrt, Heidi Lempp, Jie Li, Santosh Loganathan, Pallab K. Maulik, Ning Ma, Uta Ouali, Renee Romeo, Nicolas Rüsch, Maya Semrau, Tatiana Taylor Salisbury, Nicole Votruba, Syed Shabab Wahid, Wufang Zhang, Graham Thornicroft

**Affiliations:** 1 Centre for Global Mental Health and Centre for Implementation Science, Health Service and Population Research Department, Institute of Psychiatry, Psychology and Neuroscience, King’s College London, London, UK; 2 Centre for Implementation Science, Health Service and Population Research Department, Institute of Psychiatry, Psychology and Neuroscience, King’s College London, London, UK; 3 Department of Biostatistics and Health Informatics, Institute of Psychiatry, Psychology and Neuroscience, King’s College London, London, UK; 4 National Institute of Mental Health and Neurosciences (NIMHANS), Bengaluru, India; 5 Health Service and Population Research Department, Institute of Psychiatry, Psychology and Neuroscience, King’s College London, London, UK; 6 Care Policy and Evaluation Centre, London School of Economics and Political Science, Houghton Street, London, UK; 7 Department of Preventive Medicine, School of Public Health, College of Health Sciences, Addis Ababa University, Addis Ababa, Ethiopia; 8 Transcultural Psychosocial Organization (TPO) Nepal, Kathmandu, Nepal; 9 Centre for Global Mental Health, Health Service and Population Research Department, Institute of Psychiatry, Psychology and Neuroscience, King’s College London, London, UK; 10 Department of Psychiatry, WHO Collaborating Centre for Mental Health Research and Capacity-Building, School of Medicine, College of Health Sciences, Addis Ababa University, Addis Ababa, Ethiopia; 11 Centre for Innovative Drug Development and Therapeutic Trials for Africa, College of Health Sciences, Addis Ababa University, Addis Ababa, Ethiopia; 12 Department of Mental Health and Substance Use, World Health Organization, Geneva, Switzerland; 13 South London and Maudsley NHS Foundation Trust, London, UK; 14 Division of Global Mental Health, Department of Psychiatry and Behavioral Sciences, George Washington University, Washington, DC USA; 15 Centre for Rheumatic Diseases, Department of Inflammation Biology, School of Immunology and Microbial Sciences, Faculty of Life Sciences & Medicine, King’s College London, London, UK; 16 The Affiliated Brain Hospital of Guangzhou Medical University (Guangzhou Huiai Hospital), Guangzhou, China; 17 George Institute for Global Health, New Delhi, India; 18 Faculty of Medicine, University of New South Wales, Sydney, Australia; 19 Peking University Sixth Hospital, Peking University Institute of Mental Health, Beijing, China; 20 Department Psychiatry A, Razi University Hospital, La Manouba, Tunisia; 21 Faculty of Medicine of Tunis, University of Tunis El Manar, Tunis, Tunisia; 22 King’s Health Economics, Health Service and Population Research Department, Institute of Psychiatry, Psychology and Neuroscience, King’s College London, London, UK; 23 Section of Public Mental Health, Department of Psychiatry II, Ulm University and BKH Günzburg, Ulm, Germany; 24 Centre for Global Health Research, Brighton and Sussex Medical School, University of Sussex, Falmer, Brighton, UK; 25 Nuffield Department of Women’s & Reproductive Health, University of Oxford, Oxford, UK; 26 Department of Global Health, School of Health, Georgetown University, Washington, DC USA; 27 The George Institute for Global Health, Imperial College London, London, UK

**Keywords:** Low- and middle-income countries, Mental health, Health service research, Global health, Community mental health, Stigma, Discrimination, Intervention

## Abstract

There is increasing attention to the impacts of stigma and discrimination related to mental health on quality of life and access to and quality of healthcare. Effective strategies for stigma reduction exist, but most evidence comes from high-income settings. Recent reviews of stigma research have identified gaps in the field, including limited cultural and contextual adaptation of interventions, a lack of contextual psychometric information on evaluation tools, and, most notably, a lack of multi-level strategies for stigma reduction. The Indigo Partnership research programme will address these knowledge gaps through a multi-country, multi-site collaboration for anti-stigma interventions in low- and middle-income countries (LMICs) (China, Ethiopia, India, Nepal, and Tunisia). The Indigo Partnership aims to: (1) carry out research to strengthen the understanding of mechanisms of stigma processes and reduce stigma and discrimination against people with mental health conditions in LMICs; and (2) establish a strong collaborative research consortium through the conduct of this programme. Specifically, the Indigo Partnership involves developing and pilot testing anti-stigma interventions at the community, primary care, and mental health specialist care levels, with a systematic approach to cultural and contextual adaptation across the sites. This work also involves transcultural translation and adaptation of stigma and discrimination measurement tools. The Indigo Partnership operates with the key principle of partnering with people with lived experience of mental health conditions for the development and implementation of the pilot interventions, as well as capacity building and cross-site learning to actively develop a more globally representative and equitable mental health research community. This work is envisioned to have a long-lasting impact, both in terms of the capacity building provided to participating institutions and researchers, and the foundation it provides for future research to extend the evidence base of what works to reduce and ultimately end stigma and discrimination in mental health.

## Background

Alongside increased efforts to improve access to, and utilisation of, quality services for mental health care in low- and middle-income countries (LMIC), there is a recognition that reducing stigma and discrimination is vital for the successful delivery of care, improving quality of lives, and protecting human rights [1–3].

Stigmatisation refers to the devaluation and discrimination expressed towards, and experienced by, people affected by mental health conditions [4, 5]. Globally, experiences of negative discrimination have been reported in clinical samples by over 80% of people with either schizophrenia or major depressive disorder [6, 7]. Stigma can result in a range of negative impacts on social inclusion and wellbeing, including poor access to health care for both physical [8] and mental [9, 10] health conditions.

Considering the impact of stigma and discrimination on access to care is important in view of the treatment gap in mental health services. The treatment gap represents the difference between the true prevalence of mental illnesses, and the proportion of the people affected by mental health conditions who receive adequate treatment and care [11]. Broadly, it can be considered reflective of a range of dimensions restricting equitable access to care [12]. Worldwide, only 15–25% of persons with severe mental disorders in low-income settings received treatment [13]. In lower-middle income countries around the world, less than 5% of people with major depressive disorder receive minimally adequate treatment [14].

**Fig. 1:**
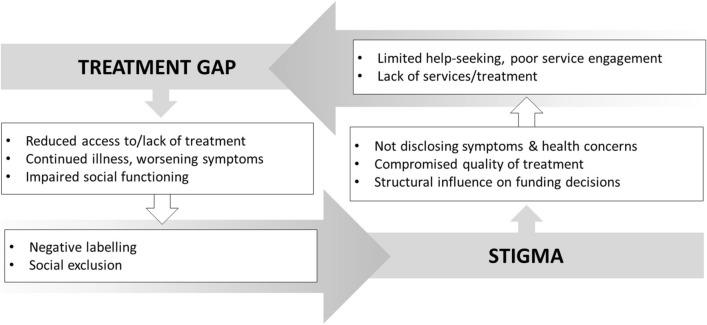
The treatment gap can be exacerbated by stigma, and vice versa

The treatment gap is both exacerbated by stigma and contributes to worsening stigma (see Fig. 1). Reduced access to care and the associated lack of treatment can result in continued illness and/or worsening symptoms. A severe mental illness or more impaired social functioning can fuel stigma by increasing negative labelling and social exclusion. Stigma and discrimination can also contribute to the treatment gap. Due to stigma, people with mental health conditions might prefer to not disclose symptoms or mental health concerns to others, including health care professionals, resulting in low rates of help-seeking or reluctance to initiate and/or continue with treatment [10, 15, 16]. This can exacerbate the impact of the treatment gap by hindering help-seeking and further reducing the chances of access to treatment, even if treatment was available. Stigma and discrimination can also contribute to the treatment gap via impacting on the quality of treatment that people with mental health conditions receive from healthcare professionals, who themselves are recognised as a key source of stigma and discriminatory behaviours [17–20]. Stigma can also have an influence on the treatment gap at a structural level, through influencing funding and investment decisions that disadvantage mental health services [21].

Scarcity of funding in mental health services is observed worldwide, but the issue is particularly pronounced in LMIC settings. LMICs allocate only between 0.5 and 1.9% of their health budgets to the treatment and prevention of mental illnesses [22], although these illnesses account for up to a third of years lived with disability and 13% of disability-adjusted life-years [23] and the World Health Organization (WHO) recommends that as a minimum 5% of the health budget is allocated to mental health [24].

Reducing stigmatisation towards people with mental illness is one of the major goals of the WHO Mental Health Action Plan [25]. In the 2017 WHO Mental Health Atlas [26] of the nearly 350 functioning prevention and promotion programmes reported by WHO Member States 40% were aimed at improving mental health literacy or combating stigma. There are burgeoning initiatives dedicated to reduction of stigma against persons with mental illness around the world, such as Time to Change (England), Opening Minds (Canada), One of Us (Denmark), beyondblue (Australia), and Time to Change Global (Ghana, India, Nigeria, Kenya, Uganda) among others [27].

Evidence-based principles for stigma reduction exist, with a consistent pattern of benefits in terms of knowledge improvement and positive attitude change from stigma reduction interventions based on inter-personal contact [1, 28, 29] albeit the available evidence could be strengthened further [30]. This approach draws on social contact theory [1, 31]; that is, reducing stigma and discrimination through facilitating intergroup contact between people with a stigmatised characteristic (in this case, mental illness) and those without. However, the evidence-base on this from LMICs is still limited [28, 32] albeit emerging [33–35].

Despite the growing interest and evidence on stigma reduction, the research on what works best, for whom, and in what settings remains limited. A state of the field collection of reviews on *Stigma Research and Global Health* identified major gaps in what has been studied and the quality of the research [36–43]. These include, for example, the gap in how stigma interventions are typically designed, implemented, and evaluated with a focus on one setting or health system level, and are limited to a particular domain of stigma [40, 41]. A second gap is the lack of systematic approaches to cultural and contextual adaptation of anti-stigma strategies implemented in diverse settings around the world [44]. The third gap is the lack of behavioural measures and valid, contextually adapted measurement tools, which limits the generation of evidence on what works in which contexts in stigma reduction.

Furthermore, successful strategies for reducing stigma and discrimination are only possible through collaboration and partnership with people with lived experience (PWLE) of mental health conditions, their families, and their advocates [39, 45, 46]. Engagement of health workers and other stakeholders who experience discrimination because of their involvement in mental health services is also vital [40]. There have also been limited initiatives to support the development of a stigma research workforce in LMICs [47]. Ultimately, equitable sustainable partnerships need to be representative of the populations receiving, delivering, and researching mental health services.

## The Indigo Partnership

The Indigo Partnership is a five-year research programme (http://www.indigo-group.org/indigo-partnership-research-programme/) that commenced in 2018, funded by the UK Medical Research Council [MR/R023697/1] [48]. The aims and objectives of the Indigo Partnership are summarised in Panel 1. The Indigo Partnership developed from the International Study of Discrimination and Stigma Outcomes (INDIGO) project—a long-standing global network of initiatives in stigma reduction [6, 7, 47].

Panel 1: Aims and objectives of the Indigo Partnership The Indigo Partnership research programme has two overall aims. The **first aim** is to carry out research to strengthen the understanding of mechanisms of stigma processes and reduce stigma against people with mental health conditions in LMICs. The **second aim** is to establish a strong collaborative research consortium through conducting this work, to undertake further joint research in the longer term.

These aims are achieved through six specific objectives:

1. Establish an active and sustainable research consortium which acts as a highly collaborative network, across institutions in various diverse cultural and contextual settings.
2. Build research capacity across the consortium, with a particular focus on early career researchers and institutions in LMICs to strengthen their capability to become centres of excellence for future multi-site intervention studies related to mental health stigma reduction.
3. Conduct formative cross-cultural research, building on literature reviews and situational analyses, to identify stigmatising language, behaviours, and institutional practices and their underlying mechanisms of action in stigmatisation processes across diverse cultural contexts.
4. Establish a harmonised online evaluation toolkit of culturally adapted and psychometrically evaluated research instruments and scales specifically designed for use in stigma-reduction intervention studies.
5. Develop and pilot the implementation of effective, contextually adapted anti-stigma interventions in LMICs settings. These interventions are based on the principle of interpersonal contact as a stigma reduction strategy, and focus on stigma reduction in community, primary care and specialist care settings. The specific mechanisms of action of these interventions are identified through formative cross-cultural research.
6. Develop further research protocols based on the results of this work, for the design and conduct of future large-scale multi-site randomised controlled trials with a focus on both effectiveness and implementation of the stigma-reduction interventions, piloted during the Indigo Partnership work.

## Setting: Indigo Partnership countries and sites

The Indigo Partnership involves research partners at seven collaborating institutions in five LMICs: China (Beijing), Peking University Sixth Hospital; China (Guangzhou), Affiliated Brain Hospital of Guangzhou Medical University; Ethiopia, School of Public Health at Addis Ababa University; India (Bengaluru), National Institute of Mental Health and Neurosciences (NIMHANS); India (Delhi), George Institute for Global Health; Nepal, National Institute of Mental Health and Neurosciences (TPO Nepal); and Tunisia, Razi University Hospital La Manouba, affiliated with the University of Tunis El Manar.

Colleagues at these institutions are leading on the practical implementation of research within the Indigo Partnership. These sites were purposefully selected to represent culturally varied settings, as well as diverse contexts in terms of health care provision and country-level economic indicators. Of these countries where the research is carried out, one represents a low-income setting (Ethiopia), three lower-middle income settings (India, Nepal, Tunisia), and one an upper-middle income setting (China) [49]. This variability across and within the implementation sites is of importance, as cultural, economic, political and health contexts influence what is stigmatised in each specific setting. By gaining insights from a broad range of contexts it is intended that the findings of the Indigo Partnership can be generalised beyond the current research sites. The sites also use major languages in LMICs (including Amharic, Arabic, Hindi, Kannada, Mandarin, and Nepali), meaning the Indigo Partnership materials are produced in widely used languages and can as such benefit large populations beyond the current study sample.

In addition to the research institutions at the implementation sites, the Indigo Partnership involves supporting research partner institutions: Brighton & Sussex Medical School (UK), London School of Economics and Political Science (UK), George Washington University (US), Ulm University (Germany), and the WHO (Switzerland). Colleagues from these institutions are leading on elements within the Indigo Partnership work through, for example, informing the development of specific research procedures, coordinating their implementation, providing technical guidance, and supporting the dissemination of the findings through dedicated knowledge exchange activities.

The overall coordination of the Indigo Partnership is led by King’s College London, UK.

**Fig. 2:**
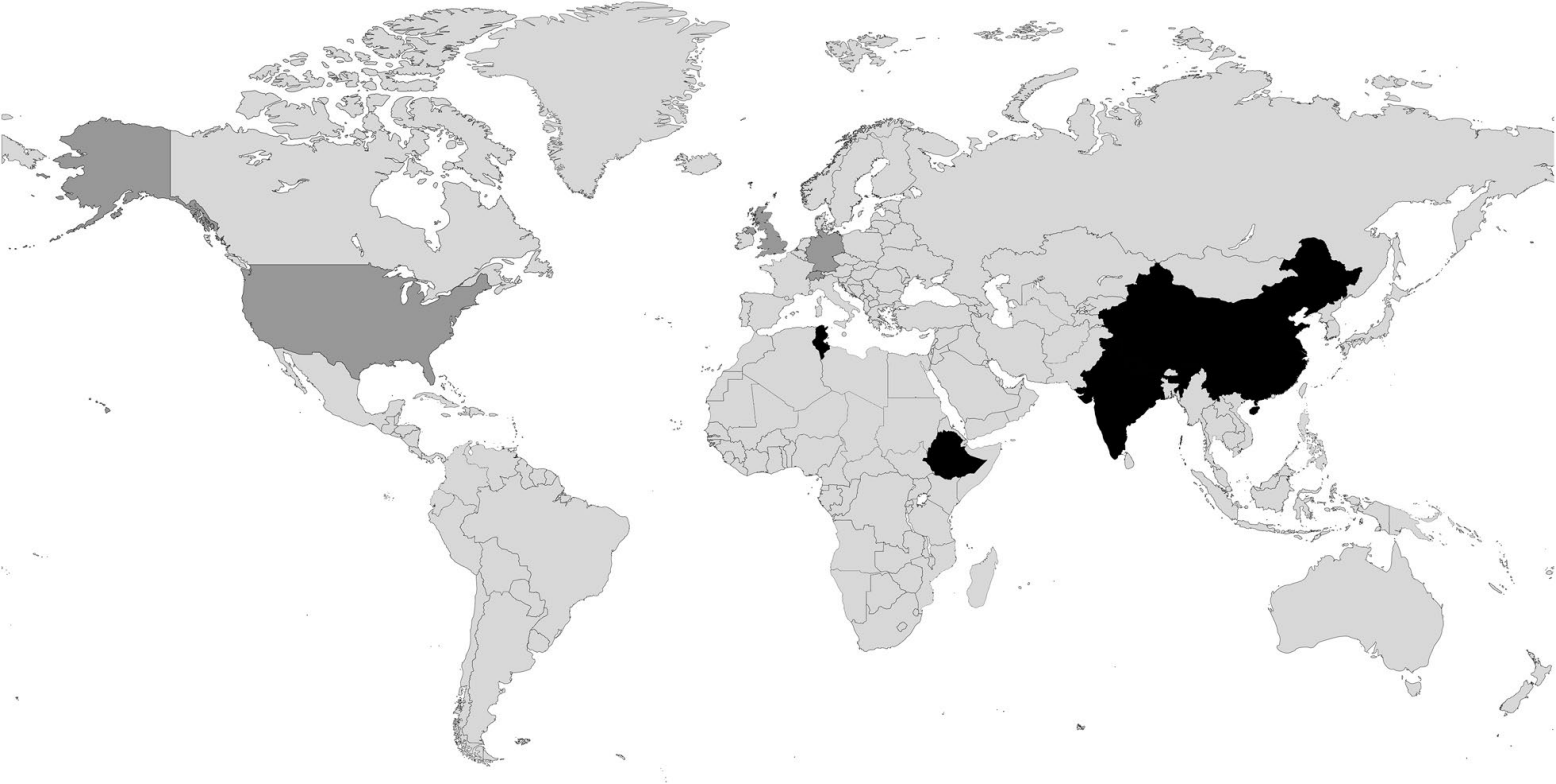
Map indicating the countries where Indigo Partnership implementing partner institutions (highlighted in black) and collaborating partner institutions (highlighted in dark grey) are located

Figure 2 shows the geographic locations of the countries where the Indigo Partnership collaborating partner institutions are located.

## Research design, methods, and structure of the work

A mixed methods design is used to achieve the aims and objectives of the Indigo Partnership programme, as outlined in Panel 1.

The inter-related activities of the Indigo Partnership are divided into seven Work Packages (WPs), with each WP being a discrete component of work within the broader programme. Within an overarching cross-project coordination WP that guides all these activities (WP1) the WPs are timed as a phase of formative work (WP2 and WP3) which informs a phase of implementing and evaluating pilot interventions (WP4, WP5, WP6). A distinct set of knowledge exchange activities are focused to ensure engagement of stakeholders during the project and maximum subsequent uptake of project outputs and outcomes (WP7). Table 1 provides the aims of the seven WPs in more detail. Protocols for the specific activities within the intervention WPs will be published separately.

**Table 1:** Work packages within the Indigo Partnership research programme

Work Package (WP)	WP aim
WP1 Coordination	Overarching project management and coordination across all research activities
WP2 Culture	Conduct formative work to comprehend cultural knowledge and insights on manifestations, mechanisms and impacts of stigma, and local healthcare contexts, and to inform scale adaptation and the development of culturally customised anti-stigma interventions
WP3 Metrics	Develop culturally adapted research instruments to assess the processes and impact of anti-stigma interventions on key domains in community, primary care, and specialist settings. Evaluate the psychometric properties of these instruments, and make them available through an online database
WP4 Local	Conduct a proof-of-principle pilot study that involves delivering and evaluating a local area public awareness-raising intervention designed to reduce stigma and discrimination and increase referrals of people with mental illness for assessment and treatment
WP5 Primary	Conduct a small proof-of-principle intervention to reduce stigma among primary health care workers in order to improve the: (1) competency of primary healthcare workers delivering mental health services; (2) quality of mental health care delivered at primary health care; and (3) experience and satisfaction of mental health service users and caregivers in primary healthcare setting
WP6 READ	Conduct a proof-of-principle training intervention to accelerate the capacity of specialist mental health service staff to respond positively to stigma and discrimination affecting service users
WP7 Uptake	Carrying out knowledge exchange activities to increase the active engagement of stakeholders in the processes of the project and support achieving the optimum uptake of Indigo Partnership research findings

**Fig. 3:**
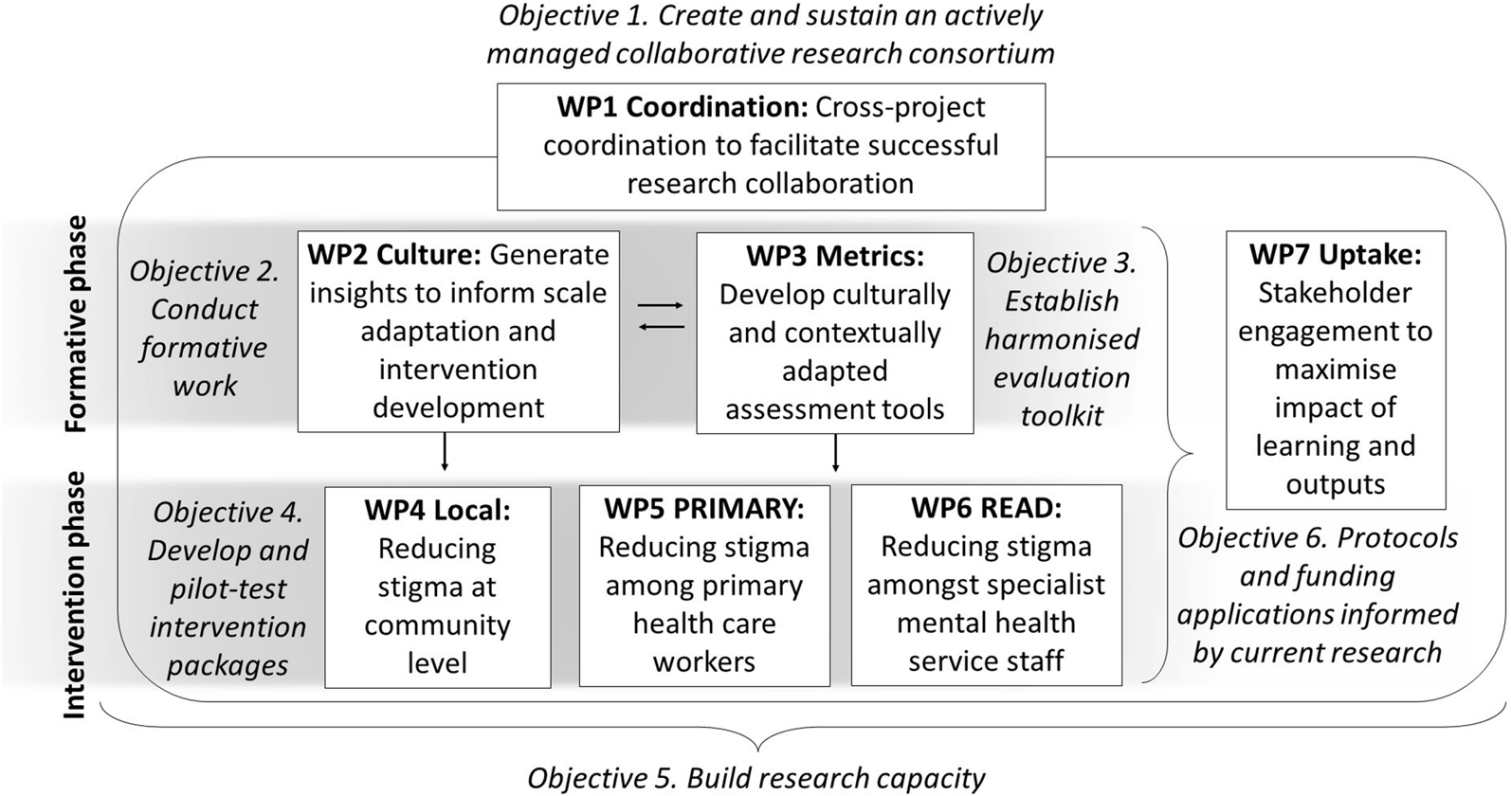
Overview of linkages between the Indigo Partnership work packages (WPs), how the work is broadly divided into phases of formative work and intervention work, and how the overall objectives of the programme (in italics) are reflected across the research elements

Figure 3 illustrates how the different WPs fit together, and how the iterative and/or sequential connections between these work components connect into a larger dynamic flow of the programme as a whole. This figure also illustrates how the practical components of work embedded within the WPs reflect the overall objectives of the Indigo Partnership, which are shown in Panel 1.

## Achieving the aims of the Indigo Partnership research programme

### Aim 1: carrying out research to strengthen the understanding of mechanisms of stigma processes and reduce stigma against people with mental health conditions in LMICs

This aim is achieved through the practical research components structured as formative (WP2 and WP3) and intervention phases of research (WP4, WP5, WP6). Overall, the formative phase uses review and qualitative methodologies to generate rich contextual insights of the research sites and involves work to develop culturally adapted research tools. These data from the formative phase inform a subsequent step to develop and pilot three anti-stigma interventions on a proof-of-principle basis. These research components address key gaps in the evidence on stigma in global health [36–43].

#### Multi-level strategy for stigma reduction

The interventions within the Indigo Partnership aim to reduce stigma and discrimination at the community, primary health care, and specialist mental health service provider levels, respectively. This work provides a harmonised package targeting a range of experiences of stigma and discrimination within community interactions, initial engagement with health care in terms of help-seeking, detection, and services delivered in primary care, as well as receipt of specialist mental health care services. The implementation sites for these interventions are carefully selected through considerations of whether the health system in a given location is able to cope with increased referrals or service demand. In addition to reducing stigma in these settings, it is envisioned that a multi-level strategy is more likely to foster structural changes in stigma, reflected in policies and resource deployment.

#### Cultural and contextual adaptation

The formative work within the Indigo Partnership explores local healthcare contexts and cultural elements of stigma, based on the concept that stigma arises from ‘what matters most’ in a cultural group [50]. These insights are integrated to the stigma-reduction activities in a systematic approach for cultural and contextual adaptation of these interventions. The learnings from this process will be compiled into a user-friendly guide for organisations and institutions wishing to likewise implement such adapted anti-stigma programmes.

#### Development of cross-cultural package of assessment tools

In response to the lack of harmonised stigma tools with established psychometric properties across diverse languages, populations, and settings, a major goal of the Indigo Partnership is to refine existing research instruments into a package that assesses stigma concepts such as social distance, knowledge, experienced discrimination, expressed stigma among health workers, as well as anticipated and observed behaviours among persons providing care and services to people with mental illness, in a culturally and contextually appropriate manner. This programme will also develop guidelines for this process so that researchers, healthcare programmers, and advocacy groups in different settings can follow the approach to develop locally adapted assessment tools.

#### Partnering with PWLE of mental health conditions

Given the evidence for stigma-reduction via social contact (32, 34, 35), the interventions within the Indigo Partnership include a key element of partnering with experts-by-experience; people who have personal experience of mental health conditions. This contact can be direct or indirect, and will be in the form, for example, of PWLE joining in stakeholder workshops, co-facilitating training and awareness-raising regarding mental illness and stigma, and sharing recovery narratives and personal testimonies [51, 52]. This partnering follows principles of non-tokenistic involvement, promoting the added value of expertise-by-experience through, for example, including PWLEs in initial stakeholder consultations alongside other key demographics in the setting, co-developing plans for PWLE involvement in the interventions, supporting PWLEs in expert positions through co-delivering training sessions, and actively working to equal power dynamics between PWLEs, members of the research teams and other stakeholders.

#### Generating insights

In addition to addressing these key gaps in the stigma literature, the work within the Indigo Partnership will also provide insights in relation to the following understudied domains: (i) mechanisms of action of stigma across cultural contexts; (ii) mechanisms of action for stigma reduction through inter-personal contact and how they operate across culture and context; and (iii) the pathways from implicit biases to explicit attitudes to behaviours and to outcomes of people with mental illness.

##### Mechanisms of action of stigma across cultural contexts

There are multiple forms of threats (e.g. fears around specific stereotypes, societal power structures, perceived health threats) that drive stigma processes based on group identities, shaping different forms and consequences of stigma [53–56]. Moreover, culture strongly influences the types of stigma that are most salient in a given setting, as prioritised values and social structures vary by context [50, 57]. Through conducting studies in seven diverse LMIC settings simultaneously with harmonised tools and approaches, this work is able to examine not only drivers of stigma, but also how culture impacts upon these processes.

##### Mechanisms of action for stigma reduction through inter-personal contact

Although inter-personal contact is known to be effective in reducing stigma [1, 29], it is less well understood under what conditions such interventions are most effective and how culture impacts upon the effectiveness of inter-personal contact in reducing stigma against persons with mental illness. Several aspects (e.g., equal status between individuals in the contact situation, or information that moderately disconfirms stereotypes) have been proposed [58–60]. The qualitative elements in the formative work inform potential facilitators of interpersonal contact and enable exploration of how inter-personal contact may operate differently based on the three intervention contexts (community, primary care, and specialist care) in the seven LMIC settings.

##### Measurement of the pathway from implicit biases to stigmatising behaviour

This research will involve the use of culturally contextualised Implicit Association Tests (IAT; an approach to measure the strength of associations between given concepts and stereotypes, assessing potential subconscious biases) and structured role play simulations to explore associations between knowledge, implicit attitudes, explicit attitudes, and health providers’ behavioural competences. These techniques offer an alternative to self-report measures and will provide insights on infrequently assessed dimensions of implicit biases and observed behaviours.

### Aim 2: establishing a strong collaborative research consortium

This aim is achieved through the following core principles underpinning all work conducted within the Partnership and activities implemented across the programme.

#### Collaborative decision-making

To establish an effective research consortium, a guiding principle of the work conducted within the Indigo Partnership research programme is a commitment to collaborative decision-making.

This is built into the programme from the outset. For example, only the broad principles of the planned work were outlined within the research proposal, and the process to operationalise these plans into actionable instructions through collective discussions is purposefully scheduled as an activity within the project. This is in a contrast to approaches where research implementation follows a pre-defined top-down research protocol.

The approach to co-production by the consortium in creating and refining detailed research protocols also ensures that expertise from across the project team, and contextual and local expertise from the research partners in the LMIC sites specifically, determines what can be achieved, and how. This way diverse practices, needs, limitations, and requirements from across the consortium are considered from the outset, with feasibility and contextual appropriateness as central considerations when refining the practical research plans.

#### Cross-project leadership

A further principle strengthening collaboration is distributing leadership roles across the project, to ensure such responsibilities are not focused solely at the core coordinating institution [61]. Each WP is led by a core group, intentionally composed of collaborators from across the project with at least one colleague from an institution in an LMIC research site. In addition to fostering collaboration, this also counters a Western-centric position in the conduct of this work. This programme follows a model which aims to actively redress and avoid power imbalances, binaries and hierarchies [62]. This principle also links to the programme’s commitment to capacity building.

#### Capacity building and mentorship

Building research capacity is a core priority within the Indigo Partnership, and also for the funder [63]. This is achieved through both specific and general activities within the programme.

Specifically, an early career researcher (ECR) mentoring scheme has been developed and is implemented for the duration of the programme. This pairs up ECRs involved with the work as mentees with senior colleague mentors with experience and/or expertise that they wish to develop (e.g., scientific communication, networking, or stakeholder engagement). Individualised career development plans are produced for the mentees, and options are identified to support the ECR’s career development goals through activities related to their role within the programme. Mentees hold an active role, to strengthen skills around establishing professional networks, taking initiative, and scheduling activities. The mentees are paired with mentors at different institutions to foster independence and provide opportunities to build professional links outside their usual networks. Further details regarding the Indigo Partnership ECR career development mentoring scheme and its evaluation will be provided in a separate publication.

In addition to the mentoring, a three-day ECR Leadership Skills Masterclass training course was arranged for junior researchers within the consortium at the onset of the programme.

Additionally, activities to support the career development of all researchers within the consortium are actively identified and encouraged. This is to ensure that collaborators at every career stage can flourish in their roles, gain skills and experience, and be competitively positioned for promotion opportunities. These opportunities have involved, for example, leadership roles within the WPs, colleagues with previous experience of primarily within-country responsibilities taking on international cross-project coordination, support with writing high-impact publications and grants, and embedding MSc and PhD research projects within this work.

#### Consortium satisfaction survey

To ensure the efforts to establish a strong collaboration do not remain purely nominal, a consortium satisfaction survey has been developed (adapted from previous work [61]). This tool provides anonymous feedback on communication, decision making, project management, and monitoring and reporting within the programme. Data is collected periodically throughout the course of the project, to actively learn how the collaboration is functioning, and crucially what can be improved.

#### Publication agreement and publication plan

For transparency and fairness regarding decision-making regarding publications arising from the Indigo Partnership research programme, a detailed publication agreement has been developed, adapted from previous work [46]. It outlines the principles for, for example, how to propose publications, sequence publication order, criteria for authorship [64], and a procedure for resolving potential disputes in relation to these decisions. This is to ensure that all collaborators have equal opportunity to contribute to publication planning, and that there is a fair distribution of first and senior authorship status amongst all who contribute to the work to actively support the career progression of early- and intermediate-career researchers.

Publications are actively monitored and supported with a publication plan, which lists all Partnership papers and their leads, writing groups, timetables and status.

## Intended impact

The impact of the Indigo Partnership is intended to be sustained through further work building on the current programme. The interventions developed and piloted through this work reflect a long-term commitment to stigma-reduction—funding applications for future international large-scale multi-site trials will be developed based on the findings. These intervention activities will be informed by insights from current implementation evaluation to maximise their acceptability, feasibility and appropriateness. Their sustainability is supported by how the intervention activities are already at the current pilot stage intended to be integrated onto existing healthcare worker training programmes or other appropriate ongoing established activities in the implementation settings. The interventions are also designed to be adapted for given local contexts through stakeholder involvement, further supporting sustainability via local buy-in. The capacity building activities are intended to strengthen the skills and experience at an individual level, but also within the collaborating research institutions, supporting their development as centres of excellence for stigma research to lead on future grant applications and subsequent work.

Insights generated through this work will be used to produce an evidence-based package of stigma reduction strategies that can be locally adapted. The knowledge exchange component of this programme is a dedicated effort to ensure maximum uptake of its findings and resources, for example, potentially as a module of the WHO Mental Health Gap Action Programme (mhGAP) Intervention Guide [65].

Sustained and widespread efforts to reduce stigma and discrimination at the community, primary and specialist care levels is envisioned to contribute towards reducing the mental health treatment gap. The procedures established through the Indigo Partnership work will support the development of standards in the area of combating stigma, contributing to global achievement of target 3.1 of the newly extended WHO Comprehensive Mental Health Action Plan 2013–2030 [25].

## Conclusions

The Indigo Partnership is a five-year research programme, with the aims: (1) to strengthen the understanding of mechanisms of stigma processes and reduce stigma against people with mental health conditions in LMICs; and (2) to establish a strong, sustainable collaborative research consortium. This work provides an important contribution to the field of mental health related stigma research, through the provision of strategies for multi-level stigma reduction, culturally and contextually adapted anti-stigma interventions, and the development of cross-cultural packages of assessment tools for stigma research. This work is envisioned to have a long-lasting impact, both in terms of the capacity building to participating institutions and researchers, and the foundation for future research to extend the evidence base of what works to reduce and ultimately end stigma and discrimination.

## Data Availability

Not applicable; manuscript does not contain any data.
